# Effect of amblyopia treatment on macular microvasculature in children with anisometropic amblyopia using optical coherence tomographic angiography

**DOI:** 10.1038/s41598-020-79585-4

**Published:** 2021-01-08

**Authors:** Tengyue Zhang, Shiyong Xie, Yangchen Liu, Caihong Xue, Wei Zhang

**Affiliations:** 1 Tianjin Eye Hospital, Tianjin Key Lab of Ophthalmology and Visual Science, Tianjin Eye Institute, Nankai University, 4 Gansu Rd, Heping Dstrict, Tianjin, 300020 People’s Republic of China; 2 Clinical College of Ophthalmology, Tianjin Medical University, Tianjin, People’s Republic of China; 3 Tianjin Eye Hospital, Tianjin, People’s Republic of China

**Keywords:** Diseases, Health care, Medical research, Signs and symptoms

## Abstract

To measure the retinal microvascular density in patients with anisometropic amblyopia using optical coherence tomographic angiography (OCTA) and to evaluate the effects of successful amblyopia treatment on microvasculature in retina. 59 children (5–12 years old) including 22 newly diagnosed unilateral anisometropic amblyopia, 16 recovered unilateral anisometropic amblyopia, and 21 control children were imaged with OCTA using 6 × 6-mm macular scan pattern. Vessel densities of the superficial capillary plexus (SCP), the deep capillary plexus (DCP), and the overall macular thickness were acquired and compared among the three groups. After adjustment for axial length, the amblyopia group showed lower macular vessel density in the SCP (*P* = 0.005) and in the DCP (*P* = 0.004) compared with that of the control group. However, for the recovered amblyopia group, no difference of vessel density was found when compared with the control group in both the SCP (*P* = 0.548) and the DCP (*P* = 0.124). No difference of the mean macular thickness was found among three groups (*P* ≥ 0.15). Children with anisometropic amblyopia have reduced macular vessel density in OCTA, while no difference of macular vessel density was found between the recovered amblyopic and control eyes. Macular thickness showed no difference in anisometropic amblyopia and remained unchanged after amblyopic treatment.

## Introduction

Amblyopia represents reduced best-corrected visual acuity (BCVA) during the years of visual development secondary to abnormal visual stimulation. Risk factors of amblyopia include uncorrected refractive error, strabismus and deprivation. With a prevalence of 0.8–3.3% among children and its lifelong and profound visual impairment consequence if without timely treatment, amblyopia has become an important public health problem around the world^1^.

Previous studies have reported morphological and functional changes in the visual cortex and lateral geniculate nucleus in subjects with various types of amblyopia^2,3^. However, whether the retina is involved in the development of amblyopia is still controversial. Numerous literatures using optical coherence tomography (OCT) have evaluated macular structural parameters, but the results are disputed^4^. More recently, the advent of optical coherence tomographic angiography (OCTA), a noninvasive in vivo neuroimaging modality, has enabled researchers to investigate the retinal microvasculature changes associated with human amblyopes^5–10^. Part of these studies have shown reduced vessel density in the macular area associated with amblyopia compared to that of fellow eyes and/or controls^5–8^, yet others reported no change of vessel density^9,10^. A few studies also indicated that mixing different types of amblyopia could confound the observations and result in inconsistency among the studies^8,10^. Additionally, the effects of successful treatment on amblyopic retinal microvasculature have not been investigated so far.

Due to the conflicting conclusions and the yet-unrecognized role of the amblyopic abnormalities at retina level, we set out to investigate the retinal vasculature in amblyopia and the effect of amblyopia treatment on retinal capillary plexus by OCTA in three groups of patients with newly diagnosed untreated unilateral anisometropic amblyopia, recovered amblyopia and age-matched and sex-matched control group to assess the retinal vascular changes and the reversibility of macular microvasculature in specific type of amblyopia.

## Results

A total of 59 patients were recruited in this study. Of those patients, 22 (37%) were newly diagnosed anisometropic amblyopia, 16 (27%) were recovered anisometropic amblyopia, and 21 (36%) were age-matched and sex-matched controls. This cohort was composed of 24 females (41%). The mean (SD) age was 7.86 (1.88) years (range 5–11 years) for patients with amblyopia, 7.75 (1.95) years (range 5–12 years) for the patients with recovered amblyopia, and 7.36 (2.22) years (range 5–12 years) for the controls. The ethnicity of all participants was Chinese. The demographic data of the patients are shown in Table 1. There was no significant difference in age and sex distribution among the three groups. The mean BCVA was 20/60 (logMAR 0.55) with a range of 20/33 to 20/200 (logMAR 0.20–1.00) in the group of amblyopic eyes. And every participant from the recovered amblyopia group and control group had BCVA of 20/20 (logMAR 0.00). As expected, the mean BCVA in the amblyopia group was significantly worse compared with that from groups of recovered amblyopia (*P* < 0.001) or control (*P* < 0.001). Moreover, the mean ± SD of spherical equivalents (SEs) was + 4.97 ± 2.58 D in the amblyopic eyes, + 4.03 ± 1.61 D in the recovered amblyopic eyes, and + 0.14 ± 1.23 D in the control eyes. Significant differences were found between the amblyopia group vs. control group (*P* < 0.001) and also the recovered group vs. control group (*P* < 0.001). No statistical difference was found in SE between the amblyopia group and recovered group (*P* = 0.210). In addition, the mean of axial length (AL) was 21.26 ± 1.11 mm in the amblyopic eyes, 21.67 ± 0.65 mm in the recovered amblyopic eyes, and 22.99 ± 0.84 mm in the control eyes. No difference was detected in mean AL between the amblyopic eyes and recovered eyes (*P* = 0.162). Comparing with the control eyes, mean AL was significantly shorter in the group of amblyopic eyes (*P* < 0.001) and in the group of recovered eyes (*P* < 0.001). The disease course and treatment process of the recovered anisometropic amblyopic children were summarized in Table 2.

**Table 1:** Patient demographics and refractive status.

Variables	AE (n = 22)	RE (n = 16)	CE (n = 21)	*P* Value		
				AE vs. CE	RE vs. CE	AE vs. RE
Age (years)	7.86 ± 1.88	7.75 ± 1.95	7.36 ± 2.22	0.423	0.578	0.857
Sex (F/M)	11/11	6/10	7/14	0.213	0.532	0.333
SE, D	4.97 ± 2.58	4.03 ± 1.61	0.14 ± 1.23	< 0.001	< 0.001	0.210
AL (mm)	21.26 ± 1.11	21.67 ± 0.65	22.99 ± 0.84	< 0.001	< 0.001	0.162
logMAR VA	0.55 ± 0.28	0.00 ± 0.00	0.00 ± 0.00	< 0.001	> 0.99	< 0.001

Unpaired *t* test was used for analysis of continuous variables, χ^2^ test was employed for analysis of categorical variables, data shown as mean ± SD.

*AE* amblyopia eyes, *RE* recovered amblyopia eyes, *CE* control eyes, *SE* spherical equivalents, *AL* axial length, *F* female, *M* male, *D* diopter, *NA* not applicable, *SD* standard deviation.

**Table 2:** Treatment summary of recovered amblyopic children.

Patient no./gender/age (years)	Age at diagnosis (years)	BCVA prior to refractive correction and occlusion (logMAR)	Duration of partial occlusion^a^ (years)	Duration of BCVA reaching 20/20^b^ (years)
1/M/6	3	0.5	0.75	2
2/M/11	6	0.7	4.5	1
3/F/5	3	0.7	2	0
4/F/6	4	0.4	2	0.5
5/M/12	5	0.4	4.5	3
6/M/7	6	0.5	1	0
7/M/9	4	0.5	4.5	1
8/F/7	5	0.4	2	0
9/F/6	3	0.7	3	0.25
10/F/7	5.5	0.5	1.75	0.5
11/M/9	7	0.2	1	1.5
12/M/9	5	0.3	4	1
13/M/6	5	0.3	1	0
14/M/8	5	0.7	3	0
15/F/7	3	0.5	4	0.25
16/M/9	4.5	0.5	3.5	1.5

^a^Together with refractive correction.

^b^Refractive correction was continued if needed.

As shown in Table 3, the mean (SD) of overall macular vessel density of superficial capillary plexus (SCP) was 48.6% ± 3.65% in the amblyopia group, 51.0% ± 2.56% in the recovered amblyopia group, and 51.0% ± 2.08% in the control group. After adjustment for AL, the amblyopia group showed a lower overall macular vessel density compared with the control eyes (*P* = 0.005) with a difference of − 3.36%. The difference was − 2.69% between the amblyopia group and recovered group (*P* = 0.007). No difference in vessel density was found between the recovered eyes and control eyes (*P* = 0.548).

**Table 3:** Macular vessel density in amblyopic eyes, recovered amblyopic eyes and control eyes.

Retinal region	Vessel density, mean (SD)%			*P* value Difference (95% CI)^a^		
	AE	RE	CE	AE vs. CE	RE vs. CE	AE vs. RE
SCP	48.6 ± 3.65	51.0 ± 2.56	51.0 ± 2.08	0.005 − 3.36 (− 5.64 to − 1.08)	0.548 − 0.67 (− 2.88 to 1.54)	0.007 − 2.69 (− 4.61 to − 0.78)
DCP	49.0 ± 6.13	50.7 ± 5.10	50.3 ± 3.04	0.004 − 5.43 (− 8.89 to − 1.72)	0.124 − 2.70 (− 6.18 to 0.77)	0.090 − 2.60 (− 5.61 to 0.42)

Unless otherwise indicated, data are expressed as mean (SD) percentage of macular vessel density.

*DCP* deep retinal capillary plexus, *SCP* superficial retinal capillary plexus, *SD* standard deviation.

^a^Difference (95% CI) and P value were adjusted for axial length.

On the other hand, the deep capillary plexus (DCP) vessel density of the amblyopic, recovered amblyopic and the control groups were 49.0% ± 6.13%, 50.7% ± 5.10% and 50.3% ± 3.04%, respectively (Table 3). After adjustment for AL, the DCP vessel density of the amblyopic eye group showed significant reduction (− 5.43%, P = 0.004) when compared with the control group. There was no significant difference between the recovered amblyopic and control groups (*P* = 0.124). We also found a decreased trend in vessel density in 6 × 6-mm scan size in patients with amblyopia when compared with that of patients with recovered amblyopia. However, this trend was not statistically different (*P* = 0.090).

The mean of overall macular thickness was 297.14 ± 9.50 μm in the amblyopia group, 293.44 ± 13.42 μm in the recovered amblyopia group and 287.52 ± 8.36 μm in the control group. No difference was found among the three groups (*P* ≥ 0.15).

## Discussion

Our current data illustrated a significantly lower vessel density of both the SCP and DCP in 6 × 6-mm macula scan in children with anisometropic amblyopia (vs. control patients). Specially, our results suggested an average reduction of 3.36% in the SCP and 5.43% in the DCP after adjustment for AL. These results are in agreement with previous studies which reported reduced vessel density in both plexuses by OCTA, although some used different region of interests (ROIs)^5–7^. However, inconsistent results have been reported regarding the macular microvasculature in amblyopic eyes^8–10^. Chen et al.^8^ reported a significant decrease of vessel density in SCP but not in DCP on 3 × 3-mm scan size of OCTA^8^. This discrepancy may be due to the middle location of DCP in the retinal layer where is more distal from the retinal arterial and choroidal circulations^11^, a larger ROI may be needed to detect the positive changes in DCP. Moreover, Araki^10^ and Demirayak^9^ found no change of vessel density in SCP and DCP and a shrink FAZ area of SCP, whereas Sobral^7^ reported an enlarged FAZ of SCP and DCP. The inconsistency among these studies could result from the mixture of types of amblyopia in these studies, which used the fellow eyes or the BCVA equal to 20/20 as normal controls instead of healthy subjects or didn’t pay special attention to differences in AL of amblyopic eyes^7,9^ which are often hyperopic and small that can cause decreased vessel density^12,13^ in the data analysis. And persistent image projection artifacts may also induce bias into the data. Therefore, our findings of significantly reduced macular vessel density in patients with anisometropic amblyopia is novel.

In this study, we demonstrate an amblyopic vascular deficiency in the retina level. Animal experiments has shown that binocular deprivation from birth or mimicking the loss of lateral geniculate neurons seen after neonatal visual cortex ablation additionally resulted in decreased ganglion cell density in central nasal retina^14^, and a permanent shrinkage in the number of synapses in the inner plexiform layer^15,16^ concurrent with a reduction in retinal ganglion cell (RGC) synaptic activity^17–21^ and receptive field size^22^. The atrophy of RGC size, the decreased number and the function degeneration of RGC could lead to a less need for a dense vasculature in the retina. That hypothesis is in line with our findings that retinal vessel density was reduced in the amblyopes.

The most interesting finding in this study is that after the BCVA of anisometropic amblyopes enhanced to the normal level by proper amblyopic treatments, there were no statistical differences of macular vessel density between the recovered amblyopic eyes and the control eyes in both SCP and DCP. This finding indicated that anisometropic amblyopic eyes with lower macular vessel density could recover to the level of healthy eyes after full correction of the refractive errors and patching. As it is known that the visual acuity loss in anisometropic amblyopes, not strabismic amblyopia, is primarily caused by grating resolution acuity and contrast sensitivity losses which are limited by factors within the primary visual system between the retina and the striate cortex^23,24^. Successful treatment can improve visual acuity and foster the redevelopment of binocularity simultaneously^25,26^. Lots of researches have demonstrated that visual experiences during the critical period modulate visual development. And deprivation of visual experiences could lead to visual impairments^27^, whereas stimulation induces enhanced visual function and results in general improvements in vision^14,28,29^. Recently, Mui^30^ reported a daily visual stimulation in the critical period enhances multiple aspects of vision through BDNF-mediated pathways in the mouse retina rather than in the brain, which indicated a potential role for therapeutic molecular targets in the retina during the critical period that result in visual enhancement. Similarly, our results also suggested that the retina is involved in the recovery process of anisometropic amblyopia.

Additionally, it is noteworthy that although the vessel density of retinal capillary plexus in recovered amblyopic eyes had reached the normal healthy level, the SEs of these eyes were still higher than the control group and no difference comparing with the amblyopic groups. The similar situation was also found in the AL comparison among the three groups. These results suggested that the ocular refractive factor and AL were not involved in this retinal microvascular reversibility in young anisometropic amblyopic children.

Furthermore, although not statistical different between the recovered and control group in two plexuses after adjustment for AL, the inter-group difference in DCP was much higher than that in SCP (− 2.70 in DCP vs. − 0.67 in SCP). These data were further corroborated by the significant difference of vessel density found in SCP between the amblyopia group and recovered amblyopia group, whereas the lack of difference in the DCP. These results suggested that the treatment effect of macular microvasculature may have a laminar difference in the anisometropic amblyopic retina. We speculate that the microvasculature injury of the DCP in amblyopia could be more severe and may need a longer time to recover compared with the SCP. According to the data of recovered amblyopic children in our clinic (Table 2), from the BCVA reached 20/20 for the first time until being enrolled in our study, the period ranged from 1 to 3 years in 44% of our patients (7 out of 16). Concerning the possible mechanism, as described in the previous study^5^, the retinal capillary plexus comprises three main planar or laminar vascular layers in the parafoveal region: SCP, DCP, and the intermediate retinal capillary plexus^11^. The DCP layer may be at a relatively higher risk of hypoxic retinal injury due to its more distal location from the retinal arterial and choroidal circulations and the greater metabolic requirement in the middle retinal layers^31,32^. Thus, the physical structure of DCP maybe account for the lagging of DCP’s reversibility in the amblyopic retina. Also, it would be of great interest to study the mechanisms of the layer recovery difference in retina during the amblyopic treatment. To investigate the potential crosstalk between SCP and DCP recovery may identify novel signaling pathways that are involved in the recovery of amblyopia and advance our understanding of this complex disorder of experience-dependent neurodevelopment.

Unlike the macular vessel system, our data showed unchanged mean macular thickness in unilateral anisometropic amblyopes and in successfully treated anisometropic amblyopic children after adjustment for the AL. Many studies using OCT have examined retinal thickness of amblyopia and evaluated the reversal of difference after treatment with varied methodologies^33–37^. It is reported studies that showed difference in retinal thicknesses tend to show increased thickness associated with amblyopia. But more recent studies regarding macular thickness reported no difference which was inconsistent with previous findings and conclusions^4^. The reports of the reversibility of increased retinal thicknesses in amblyopic eyes with treatment were still very conflicting. Therefore, the evidence across OCT studies of association between the retinal thickness and amblyopia remains elusive.

Nonetheless, there are some limitations in our study. Firstly, it is necessary to consider that while the main criteria governing in our paper whether amblyopia treatment is ‘successful’ is BCVA, several other functional visual deficits in amblyopia may persist^4^. Whether those deficits are related to the retina vascular system needs further investigation. Secondly, as only a small number of children that enrolled from a cross-sectional design were included in this study, it is unclear whether our findings would be generalizable to longitudinal studies in a larger group of patients.

In conclusion, children with anisometropic amblyopia have reduced superficial and deep retinal capillary density on OCTA, and no difference of macular vessel density was found between the recovered amblyopic and control eyes. The reversibility of this retinal microvasculature in amblyopia may have laminar difference between SCP and DCP. This study indicated that the retinal microvasculature is involved in amblyopia and the recovery process of anisometropic amblyopia. And these results will raise a future interest and will advance our understanding of the retinal changes in amblyopia.

## Methods

This observational cross-sectional study followed the tenets of the Declaration of Helsinki for research involving human subjects and was approved by the Ethics Board of Tianjin Eye Hospital. This study was designed and conducted at Tianjin Eye Hospital from May 1, 2019, to September 30, 2019. Signed parental informed consent forms were obtained before examinations were performed on the children.

Three cohorts of newly diagnosed and untreated unilateral anisometropic amblyopia, recovered unilateral anisometropic amblyopia, and age-matched and sex-matched normal control children were recruited. All the participants were between 5 and 12 years old. Snellen BCVA was converted to logMAR for analysis. Amblyopia was defined as the interocular difference ≥ 0.2 logMAR visual acuity. Anisometropia was diagnosed as a refractive error difference of ≥ 2 diopters (D) spherical equivalent^1^. Recovered unilateral anisometropic amblyopia was defined as the interocular difference in BCVA between the sound eye and amblyopic eye became less than one line after prescribing glasses to fully correct refractive errors combined with partial occlusion therapy. Age-matched controls were enrolled from children who presented at our pediatric clinic for routine eye examination and had spherical or cylinder error < 1 D, BCVA was equal to or better than 20/20 in both eyes. The right eyes only were selected for analysis. Refraction data were converted into spherical equivalents (SEs), calculated as the spherical dioptric power plus one-half of the cylindrical dioptric power.

All children in the study underwent a comprehensive ophthalmologic examination including slit-lamp biomicroscopy, fundoscopy, subjective refraction completed after cycloplegia, and an orthoptic evaluation (Hirschberg and cover tests, and extraocular movement evaluation). Central fixation was determined by ophthalmoscopy. AL was measured by AL-Scan (NIDEK Co., Gamagori, Japan). Exclusion criteria for this study included combined mechanism amblyopia, children with a history of prematurity, systemic diseases or neurologic diseases, preexisting ocular abnormalities such as glaucoma or retinal disorders, and nystagmus. Besides, insufficient cooperation during OCTA measurements was excluded.

**Figure 1:**
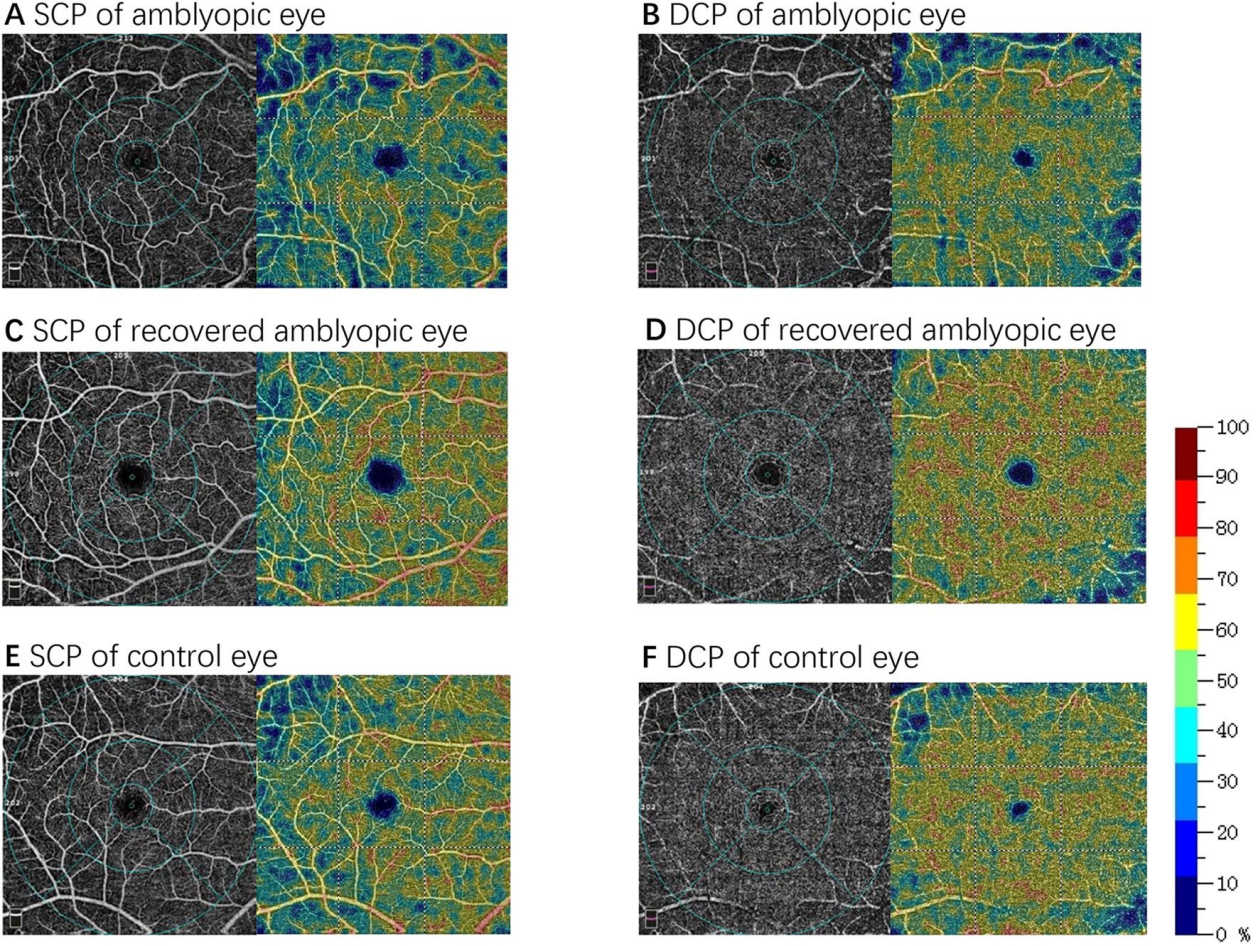
Results from 6 × 6-mm macular scans of vessel density in the superficial retinal capillary plexus (SCP) and deep retinal capillary plexus (DCP) of an amblyopic child (**A** and **B**), a recovered amblyopic child (**C** and **D**) and a control child (**E** and **F**). In the perfusion density maps, a decrease in vessel density is indicated by colder (bluer) colors.

Macular OCTA scans were performed using a spectral-domain device with built-in optical software (AngioVue version 2017.1.0.155; Optovue, Inc.). The parameters of the flow areas in the fovea-centered 6 × 6-mm scan size was measured (Fig. 1). Vessel density (as a percentage) at both the SCP and the DCP levels together with the central macular thickness from the inner limiting membrane to the retinal pigment epithelium were acquired. The 3D projection artifacts removal algorithms were implemented in the updated optical software (Optovue, Inc.) to remove projection artifacts in the DCP. All images were captured and assessed by experienced retinal specialists to ensure correct segmentation and sufficient image quality (quality index ≥ 8). Poor-quality scans were excluded from the analysis.

Study population demographics were summarized using traditional descriptive methods. A one-way analysis of covariance (ANCOVA), which was controlled using AL, was used to evaluate the differences between the three groups. Statistical analyses were performed with statistical software (SPSS version 25.0; SPSS Inc, Chicago, IL, USA). A two-sided P-value ≤ 0.05 was considered to be statistically significant.
